# The Effects of Lysophosphatidic Acid on The Incidence of Cell Death in Cultured Vitrified and Non-Vitrified Mouse Ovarian Tissue: Separation of Necrosis and Apoptosis Border

**DOI:** 10.22074/cellj.2018.5180

**Published:** 2018-05-28

**Authors:** Neda Abedpour, Mojdeh Salehnia, Nassim Ghorbanmehr

**Affiliations:** 1 Department of Anatomy, Faculty of Medical Sciences, Tarbiat Modares University, Tehran, Iran; 2 Department of Biotechnology, Faculty of Biological Sciences, Alzahra University, Tehran, Iran

**Keywords:** Cell Death, *In Vitro* Culture, Lysophosphatidic Acid, Vitrification

## Abstract

**Objective:**

The aim of the present study was to examine whether lysophosphatidic acid (LPA) could decrease cell death and improve *in vitro* culture (IVC) conditions in cultured vitrified mouse ovarian tissue.

**Materials and Methods:**

In this experimental study, we collected and randomly divided 7-day-old mouse ovarian tissues into vitrified and non-vitrified groups. The ovaries were cultured in the presence and absence of LPA for one week. Morphology and follicular development were evaluated by hematoxylin and eosin (H&E) and Masson’s trichrome (MTC) staining. The incidence of cell death was assessed by flow cytometry using annexin V/propidium iodide (PI) and a caspase-3/7 assay in all studied groups.

**Results:**

The vitrified groups had a significantly decreased follicle developmental rate compared to the non-vitrified groups (P<0.05). Overall, qualitative and quantitative results showed prominent follicular degeneration in the vitrified groups compared with the respective non-vitrified groups. Both LPA treated groups had a significantly higher proportion of preantral follicles compared to the non-LPA treated groups (P<0.05). Flow cytometry analysis results showed significantly greater early and late apoptotic cells in all groups (17.83 ± 8.80%) compared to necrotic cells (7.97 ± 0.92%, P<0.05). The percentage of these cells significantly increased in the vitrified groups compared with non-vitrified groups. LPA treated groups had a lower percentage of these cells compared to non-LPA treated groups (P<0.05). The lower enzyme activity was observed in non-vitrified (especially in the LPA^+^ groups) cultured ovaries compared to the vitrified group (P<0.05).

**Conclusion:**

Both vitrification and IVC adversely affected cell survival and caused cell death. We postulated that LPA supplementation of culture medium could improve the developmental rate of follicles and act as an anti-cell death factor in non-vitrified and vitrified ovarian tissues. It could be used for in vitro maturation of ovarian tissue.

## Introduction

Cryopreservation of ovarian tissue is the most commonly used technique ongoing improvement to preserve fertility potential in pre-pubertal girls and young women under anticancer treatments (1-8). 

*In vitro* culture (IVC) of recovered ovaries following cryopreservation is a proposed approach to support follicular development in the mammalian ovary (8-11). 

Despite some improvement in the *in vitro* mouse ovarian tissue culture (6, 12, 13), several studies have demonstrated that both cryopreservation and ovarian tissue culture affected the survival and developmental rates of follicles; however, these techniques caused an increase in the incidence of cell death in follicular and theca cells (8, 14-18). 

Cell death induced by physical and chemical conditions (19) during cryopreservation and/or IVC impacts the quality, growth, survival, and development of ovarian follicles (20, 21). The pattern of this cell death is not properly identified and may be attributed to apoptosis or necrosis. The knowledge about necrosis and apoptosis incidence in vitrified-cultured ovaries could direct us to improve culture conditions and cryopreservation techniques. 

Common inducers of apoptosis and necrosis include oxidative stress, protease activation, and hypoxia (22-24).

Numerous attempts have been made to improve IVC conditions by the addition of growth factors, antioxidants, and anti-apoptotic factors (9, 24-26).

Lysophosphatidic acid (LPA) is a natural, ubiquitous lysophospholipid (molecular weight: 430-480 Da) normally found in various tissues such as the testes, ovaries, and follicular fluid (27). LPA participates in cell survival, migration, proliferation, differentiation, and cell-to-cell interactions (28, 29). It contributes to follicular activation and oocyte growth in vivo (24-26, 30). In bovines, LPA has been demonstrated to stimulate the expression ratio of oocyte quality marker genes. In addition, supplementation of oocyte maturation medium with 10^-5^ M LPA promoted an anti-apoptotic balance that resulted in a significantly higher BCL2/BAX ratio (25). Jo et al. (24) demonstrated that supplementation of oocyte *in vitro* maturation medium with 30 µM of LPA had a positive effect on the developmental competence of mouse oocytes without a detrimental effect on spindle normalcy or mitochondrial integrity, and did not affect the apoptosis rate.

According to the best of our knowledge, there is scant information regarding the effects of LPA on in vitro follicular development and incidence of cell death. Thus this study has been designed to evaluate follicular development followed by the incidence of cell death (necrosis and apoptosis) during 7 days of IVC of mouse ovaries in the presence and absence of LPA by flow cytometry using annexin V/propidium iodide (PI) and a caspase-3/7 assay. 

## Materials and Methods

### Animals and ovarian tissue

In this experimental study, we collected ovarian tissue from 7-day-old National Medical Research Institute (NMRI) mice. The mice were housed under a 12 hour light/dark cycle and controlled temperature (22 ± 2°C) in the animal house of the Tarbiat Modares University. Animals were handled according to the Ethical Guidelines for the Care and Use of Laboratory Animals and protocols set by Tarbiat Modares University (ref. no: 52/8188). The mice (n=78) were sacrificed by cervical dislocation. Their ovaries were isolated and dissected mechanically, and placed in alpha-minimum essential medium (α-MEM, Gibco, UK) supplemented with 5% fetal bovine serum (FBS, Gibco, UK), penicillin (Gibco, UK), streptomycin (Gibco, UK), sodium pyruvate (Sigma, USA), and sodium bicarbonate (Sigma, USA) until use. 

### Experimental design

We randomly divided the ovaries into two groups, non-vitrified (n=78) and vitrified (n=78). The non- vitrified and vitrified groups were further subdivided. The subgroups consisted of whole ovaries not cultured (n=26 per subgroup) and those cultured for one week in α-MEM medium in the presence and absence of LPA (n=52 per subgroup). Hence, the study design consisted of 6 groups: non-vitrified, vitrified, non- vitrified LPA^-^, non-vitrified LPA^+^, vitrified LPA^-^, and vitrified LPA^+^. All groups underwent the following assessments: morphological study with hematoxylin and eosin (H&E) and Masson’s trichrome (MTC) staining, and flow cytometry analysis with annexin V/ PI and a caspase-3/7 assay. 

### Vitrification and warming procedures

Whole ovaries were vitrified according to a previously published protocol (31) with slight modifications. Briefly, the ovaries (n=78) were transferred into vitrification medium (EFS40) that contained 40% ethylene glycol (v/v), 30% Ficoll 70 (w/v), and 1 mol sucrose for 5 minutes at room temperature. Then, the ovaries were individually loaded onto a Cryolock® (Biotech, USA), immersed in liquid nitrogen, and stored for one week. For warming, we placed each of the Cryolocks in 1000 µl of descending concentrations of sucrose (1, 0.5, 0.25 M) for 5 minutes at room temperature. Warmed ovaries were incubated for 30 minutes in α-MEM media supplemented with 5% FBS prior to evaluation.

### *In vitro* culture of ovarian tissues 

Non-vitrified and vitrified ovaries in the presence and absence of 20 µM LPA (n=26 per subgroup) (24) were cultured individually on culture inserts (Millicell^®^ CM, 0.4 µm pore size, Millipore Corp., Billerica, MA, USA) in 24-well plates that contained 400 µl of α-MEM medium supplemented with 1% insulin, transferrin, and selenium (ITS, Gibco, UK), 10% FBS, and 100 mIU/ml recombinant FSH (rFSH or Gonal-F, Serono, Switzerland) in a humidified incubator with 5% CO_2_ at 37°C for 7 days. Half of the culture media (0.2 ml) was refreshed every other day and the rest was collected and stored for hormonal analysis. During the culture period the morphology of ovaries was observed and evaluated under an inverted microscope.

### Histological evaluation

Morphological and histological examinations of all studied groups (n=5 in each subgroup) were assessed by H&E staining before and after IVC. The ovaries (n=30 in total) were fixed in Bouin’s fixative for 6-8 hours and dehydrated through an ethanol series (70-100%), immersed in xylol, and subsequently embedded in paraffin. The paraffin embedded tissues were serially sectioned into 5 µm thicknesses and mounted on slides with 5 interval, and stained with H&E. Finally, we performed field by field assessments of tissue morphology and the numbers of ovarian follicles under a light microscope. The normal ovarian follicles were determined and classified as follows: i. Primordial follicles that contained immature oocyte with a single layer of flattened granulosa cells, ii. Primary follicles that had a single layer of cuboidal granulosa cells, and iii. Preantral follicles that were surrounded by two or more layers of cuboidal granulosa cells (32). To avoid duplicate counting of the follicles, we only counted the follicles that contained a visible nucleus in the oocyte. 

### Masson’s trichrome staining

Another set of serial sections with 5 µm thickness and 5 intervals was stained with MTC and evaluated for stromal tissue morphology under a light microscope (n=3 per subgroup). 

### Ovarian area

We assessed ovarian morphology under an inverted microscope every 48 hours during the culture period on days 3, 5, and 7 in all of the studied groups (n=5 per subgroup). Photos of each ovary at the same magnification were captured and analyzed by Digimizer software (MedCalc Software bvba). The area of each ovary was measured in units of pixels and converted to millimeters. Next, we calculated the surface area of each ovary (µm2) by using this software.

### Flow cytometry

Flow cytometry analysis was done in all study groups to detect intact, apoptotic, and necrotic cells. The ovaries (n=9 per subgroup in three repeats) were dissociated mechanically by pipetting, then put in collagenase I solution (800 IU/ ml) and incubated for 15 minutes. The cell suspension was filtered through a 100 µm nylon mesh cell strainer and incubated for 15 minutes. Then, the suspension was washed twice with warm PBS. The cell suspensions (10^6^ cells/ml) were incubated for 15 minutes in annexin V-fluorescein isothiocyanate (FITC) and PI staining solution according to the kit’s instructions (Annexin V-FITC Apoptosis Detection Kit, Biotool, UK). Finally, we added the binding buffer. Early apoptotic cells had a green fluorescence, whereas late apoptotic cells showed orange fluorescence, necrotic cells had red fluorescence, and intact cells did not show any fluorescence. All fluorescence activated cell sorting (FACS) data were analyzed with FlowJo software (Life Sciences, Ashland, OR, USA).

### Caspase-3/7 activity assay 

We assessed the concentration of caspase-3/7 activity in ovarian tissues (n=9 per subgroup in three repeats) according to the Caspase-Glo^®^ 3/7 Assay Kit (Promega, Madison, WI, USA). The ovaries were homogenized (n=9 per subgroup in 3 repeats) in 200 µl hypotonic extraction buffer with 25 mM HEPES (pH=7.5), 5 mM MgCl2, and 1 mM EDTA. Then, the products were sedimented by centrifugation at 5000×g for 15 minutes at 4°C, and the supernatants were used for the assay. The total protein level of each sample was detected by the Bradford method (Bio-Rad).

Diluted (10 µg/ml) extract was mixed with Caspase- Glo® reagent and incubated at 37°C for 60 minutes for detection of caspase-3/7 activity. The extract was subsequently placed in a Sirius Single Tube Luminometer (Berthold Detection Systems GmbH, Germany) and measured in terms of relative light units (RLU, Berthold LB9501 luminometer). Finally, we determined caspase-3/7 activity per 1 mg/ml of protein.

### Statistical analysis

Statistical analysis was performed with SPSS program version 21 (SPSS Inc., USA) software. Values are given as mean ± SD. The data of follicular count, ovarian area, flow cytometry, and caspase-3/7 activity were compared with the one-way ANOVA and post hoc Turkey’s tests. A P<0.05 was considered statistically significant at the 95% confidence level. 

## Results

### Phase contrast morphology of cultured ovaries 

**Fig.1:**
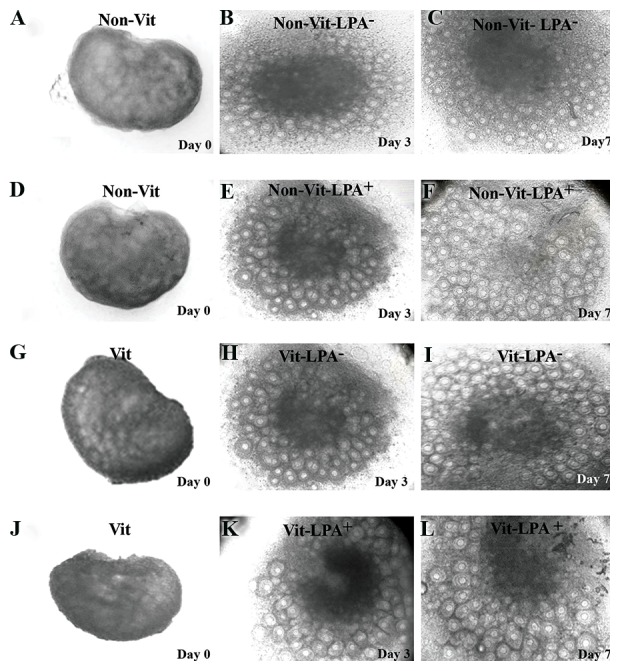
Photomicrographs of mouse ovaries viewed under an inverted microscope. A-C. Non-vitrified in the absence of lysophosphatidic acid (LPA), D-F. Non-vitrified in the presence of LPA, G-I. Vitrified in the absence of LPA, and J-L. Vitrified in the presence of LPA (scale bar: 100 µm).

The morphological changes of the ovarian tissues according to phase contrast microscopy in all of the studied groups were shown in Figure 1. Our results demonstrated that at the end of culture period the follicles exhibited outgrowth in the margin of the ovaries. This finding was more visible in the non- vitrified LPA^+^ growth. The central parts of all cultured ovaries were dark; these dense areas were more prominent in the vitrified LPA^-^ group (Fig .1).

### Light microscopy observation

**Fig.2:**
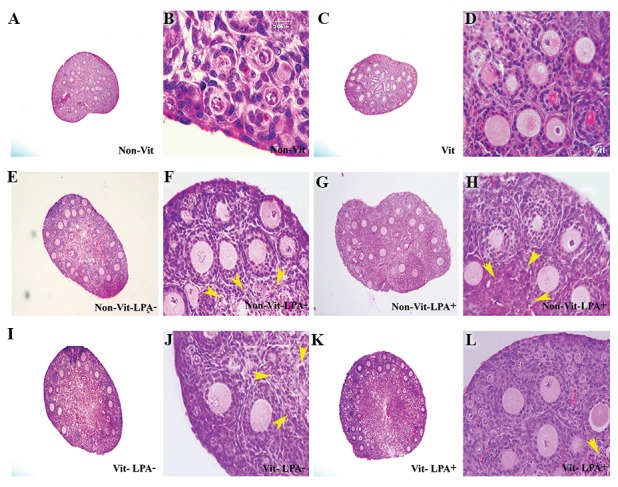
Photomicrographs of mouse ovarian tissues viewed under the light microscope using haematoxylin and eosin staining. A. Non-vitrified whole ovaries using low magnification, B. High magnification of several primordial follicles from the non-vitrified ovaries, C. Vitrified whole ovaries using low magnification, D. High magnification of the vitrified whole ovaries group, E, F. Non-vitrified ovary in the absence of LPA, G, H. Non-vitrified ovary in the presence of LPA, I, J. Vitrified ovary in the absence of LPA, K and L. Vitrified ovary in the presence of LPA. The follicles that were at different stages of development with normal and atretic morphology were seen on 7th day of culture in non-vitrified and vitrified ovaries, damaged follicles with pyknotic nuclei of oocytes and irregular shape of granulosa cells were prominent in the central parts of cultured ovaries (arrow heads) (scale bar: 200 µm).

The histological morphology of the ovaries in studied groups that were stained with haematoxylin and eosin are presented in Figure 2. The cortical parts of the cultured ovaries demonstrated the normal appearance of follicles that contained an oocyte with germinal vesicle and granulosa cells. Close adhesion was seen between the oocyte and granulosa cells in follicles at different developmental stages. The central parts of the cultured ovaries had damaged follicles with pyknotic nuclei of the oocytes and irregularly shaped granulosa cells.

### Percent of normal follicles in the studied groups

The overall quantitative results of the numbers of normal follicles at different developmental stages in all study groups of study were summarized in Table 1.

Before the IVC, the proportion of primordial follicles was more than the other stages of follicular development. During the culture period, primordial and primary follicles grew to preantral follicles in all groups so the percentage of preantral follicles significantly increased after one week culture within each group (P<0.05). However, there was a significant decrease in the percent of preantral follicles observed in both vitrified groups in comparison with their respective (LPA^-^ or LPA^+^) non-vitrified samples (P<0.05). In both LPA treated groups, the proportion of preantral follicles was significantly higher than non-LPA treated groups (P<0.05). 

### Masson’s trichrome staining

**Fig.3:**
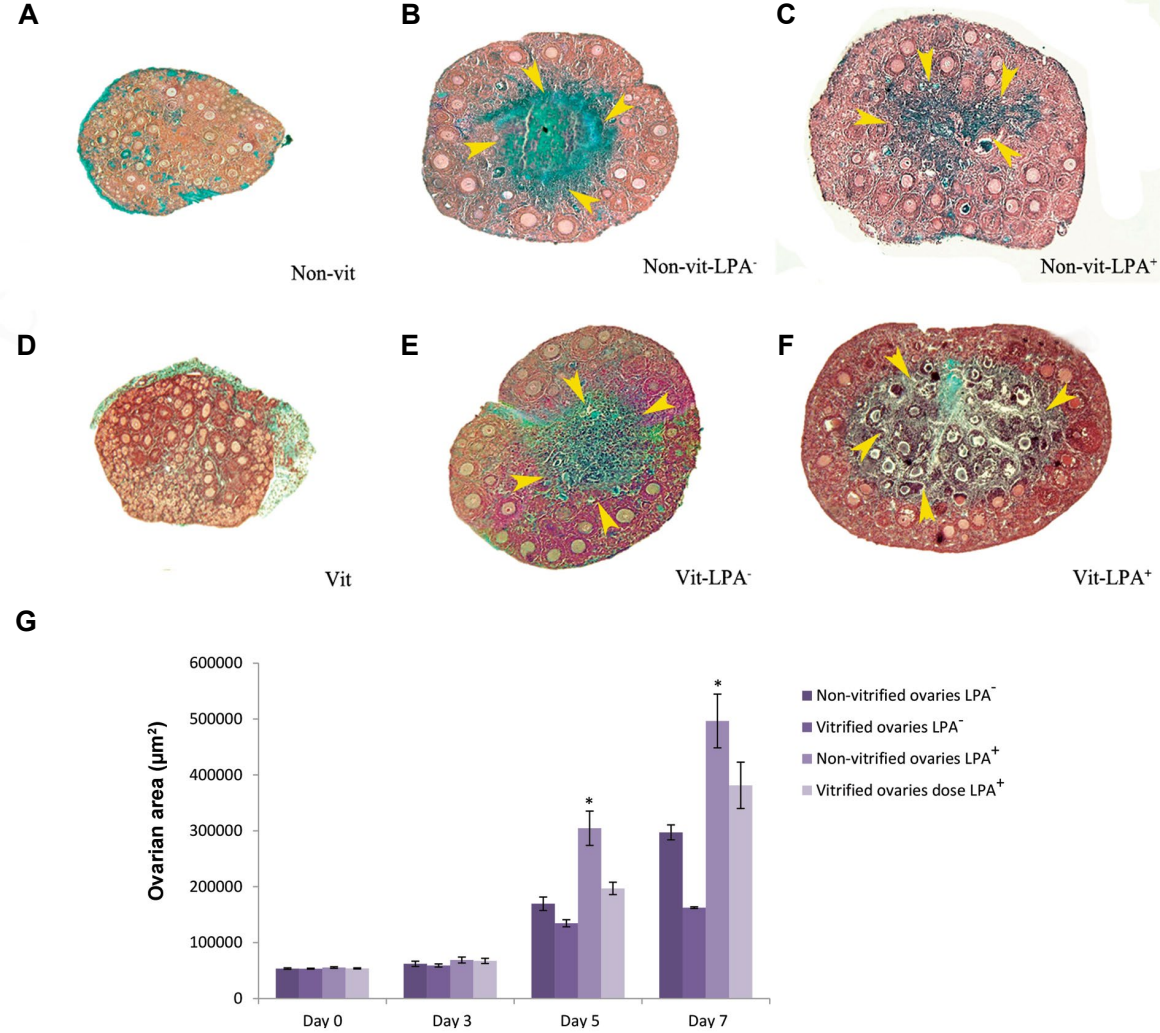
Photomicrographs of vitrified and non-vitrified whole mouse ovary sections using Masson’s trichrome (MTC) staining before and after 7 days of culture. A. Non-vitrified ovary, B. Non-vitrified ovary in the absence of lysophosphatidic acid (LPA), C. Non-vitrified ovary in the presence of LPA, D. Vitrified ovary, E. Vitrified ovary in the absence of LPA, F. Vitrified ovary in the presence of LPA. The central area of the cultured ovaries were seen as a green color all study groups that included degenerated follicles with irregularly shaped nuclei. Changes to this central area were markedly high in the vitrified LPA^-^ group and the smallest in the non-vitrified LPA^+^ group. The mean area of mouse cultured vitrified and non-vitrified ovaries on days 0 (beginning), 5, and 7 of the culture period, and G. The analysis of surface area of the ovarian tissues derived from vitrified and non-vitrified groups during culture period. *; Significant differences with other groups after culture (P<0.05) (scale bar: 200 µm). The yellow arrow head in different parts of figures showed the fibrotic and degenerated area in the center of ovarian tissue.

The morphology of the ovarian tissues according to MTC staining in all groups of study is shown in Figure 3. Before the IVC, the green-stained collagen fibers were located in the peripheral area of the ovarian tissue as tunica albuginea. After in vitro culturing, the central area of the cultured ovaries had more fibrosis according to the increased green color. Follicular degeneration (fragmented nuclei) as a result of cell death was seen in both culture groups. However, these areas were prominent in vitrified groups compared to their respective non-vitrified groups (Fig .3).

**Table 1:** Percent of follicles at different developmental stages from all studied groups


Group	Normal follicles (n)	Degenerated follicles n (% ± SD)	Primordial follicles n (% ± SD )	Primary folliclesn (% ± SD )

Non-vitrified	1020	135 (11.84 ± 0.41)	920 (90.20 ± 1.5)	39 (3.82 ± 1.3)
Vitrified	1005	206 (17.29 ± 1.2)	902 (89.75 ± 1.72)	35 (3.48 ± 0.23)
Non-vitrified LPA^-^	1465	301 (17.04 ± 0.63)	990 (67.57 ± 1.20)	131 (9 ± 1.51)
Non-vitrified LPA^+^	1317	255 (16.22 ± 2.59)	674 (51.17 ± 2.10)	152 (11.55 ± 1.90)
Vitrified LPA^-^	1263	521 (41.25 ± 2.98)^a^^,^^b^	917 (72.60 ± 1.10)	149 (11.80 ± 1.84)
Vitrified LPA^+^	1335	365 (27.34 ± 2.20)^a^^,^^b^^,^^c^	793 (59.41 ± 1.45)	147 (1.01 ± 1.31)


LPA; Lysophosphatidic acid, ^a^; Significant differences with non-vitrified ovaries before culture (P<0.05), ^b^; Significant differences with non-vitrified LPA^-^ ovaries (P<0.05), and ^c^; Significant differences with vitrified LPA^-^ ovaries (P<0.05). The percentage was calculated based on the normal follicles.

### Surface area of the ovaries

Our results demonstrated (Fig .3) a significant increase in the surface area in all studied groups during the culture period compared to the first day of culture (P<0.05). This parameter was significantly lower in the vitrified groups compared to their respective non-vitrified groups on days 5 and 7 of culture (P<0.05). In the two LPA supplemented groups, the mean surface area of the ovaries significantly increased in comparison with their control (P<0.05). 

### Flow cytometry 

**Fig.4:**
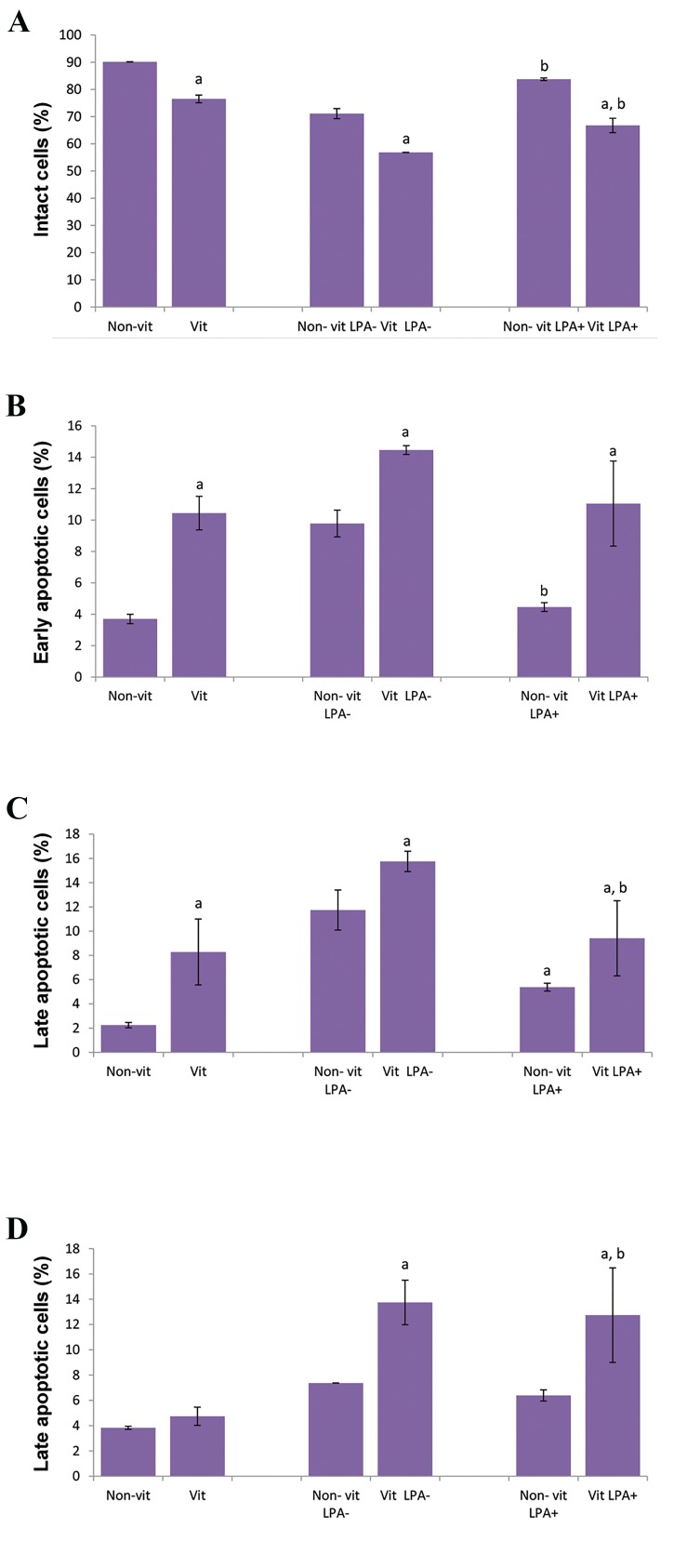
Flow cytometry analysis of ovarian tissues derived from vitrified and non-vitrified groups before and after 7 days of culture according to annexin V and propidium iodide (PI) staining. A. The percent of intact cells, B. The percent of early apoptotic cells, C. The percent of late apoptotic cells, and D. The percent of necrotic cells. ^a^; Significant differences with vitrified ovaries (P<0.05) and ^b^; Significant differences with lysophosphatidic acid (LPA)-ovaries (P<0.05).

Flow cytometry analysis showed that the percentage of intact cells was 90.14 ± 0.03% (non-vitrified), 76.52 ± 1.4% (vitrified), 71.1 ± 1.86% (non-vitrified LPA), 83.75 ± 0.47% (non-vitrified LPA^+^), 56.82 ± 0.03% (vitrified LPA), and 66.78 ± 2.68% (vitrified LPA^+^). There were significant differences between non-vitrified and vitrified groups in all areas of the study (Fig .4). The percentage of early apoptotic cells significantly decreased in the non- vitrified LPA^+^ (9.78 ± 0.85%) and vitrified LPA^+^ (4.46 ± 0.28%) compared to the non-vitrified LPA^-^(14.46 ± 0.28%) and vitrified LPA^-^ (11.05 ± 2.71) groups.

The highest percentages of late apoptotic (15.75 ± 0.84%) and necrotic (12.74 ± 3.74%) cells were in the vitrified LPA^-^group compared to 11.74 ± 1.65% apoptotic cells and 7.37 ± 0.01% necrotic cells in the non-vitrified LPA^-^group (P<0.05). However, in the two LPA treated groups, these percentages were significantly less than the non-LPA treated groups (P<0.05). 

Totally, the proportion of apoptotic cells (17.83 ± 8.80%) in all study groups were significantly higher than necrotic cells (7.97 ± 0.92%, P<0.05). 

### Caspase-3/7 activity 

Caspase-3/7 activities per milligram of protein were 1142.53 ± 86.76 RLU (non-vitrified), 1539.38 ± 94.43 RLU (vitrified), 3048.24 ± 132.99 RLU (non-vitrified LPA), 2105.32 ± 0.76 RLU (non-vitrified LPA^+^), 4030.71 ± 40.92 RLU (vitrified LPA), and 3062.95 ± 67.16 RLU (vitrified LPA^+^) as seen in Table 2. Significant differences were found between the caspase-3/7 activities in both cultured vitrified and non-vitrified groups (P<0.05). The two groups supplemented with LPA had significantly lower enzyme activity than their respective control groups (P<0.05). 

**Table 2:** Caspase-3/7 activity in all studied groups


Group	Caspase-3/7 activity (RLU/mg protein)

Non-vitrified	1142.53 ± 86.76
Vitrified	1539.38 ± 94.43
Non-vitrified LPA^-^	3048.24 ± 132.99
Non-vitrified LPA^+^	2105.32 ± 0.76^a^
Vitrified LPA^-^	4030.71 ± 40.92^a^
Vitrified LPA^+^	3062.95 ± 67.16^b^^c^


LPA; Lysophosphatidic acid. The concentration of caspase-3/7 in vitrified and non-vitrified ovaries before and after culture in the presence and absence of LPA, ^a^; Significant differences with non-vitrified LPA^-^(P<0.05), ^b^; Significant differences with vitrified LPA^-^(P<0.05), and ^c^; Significant differences with non-vitrified LPA^+^ (P<0.05).

## Discussion

This study was the first to evaluate the effects of LPA supplementation of ovarian tissue culture media on follicular development and incidence of cell death. We assessed these effects in both vitrified and non-vitrified samples. 

Our morphological observations with H&E and MTC staining indicated enhanced follicular development from the primordial follicle to the preantral stage in parallel with an increase in mean surface area of ovarian tissue in all LPA treated groups. These results demonstrated that LPA proliferated the follicular cells, not only in the non- vitrified but also in vitrified groups. The similar biological effects of LPA on cell proliferation have been previously shown in ovarian cells (33) by binding to its receptors and its involvement in the mitogen-activated protein kinase (MAPK)/p38 and phosphoinositol 3 kinase (PI3K)/Akt pathways (34).

Related reports postulated that LPA was involved in cell survival, cell activation of the entire primordial follicle pool, and promotion of nuclear and cytoplasmic maturation of mouse oocytes via its receptor (24-26, 35).

A number of studies reported cryopreservation by using vitrification methods and ovarian tissue culture significantly increased the incidence of follicular cell death (17, 36).

Abdi et al. (37) stated that vitrified neonate ovarian tissues showed lower developmental competency of follicles than non-vitrified ovaries. However, other reports revealed that the development of culture preantral follicles derived from vitrified ovarian tissue did not significantly differ from fresh samples (38, 39). The reason for this discrepancy could be related to the different tissue culture systems.

Another objective of this study was to analyze the beneficial effects of LPA on the improvement of ovarian culture by reducing the incidence of cell death. For this purpose, we employed several complementary techniques in addition to morphological staining. 

Morphological observation (H&E and MTC) showed signs of follicular degeneration and cell fragmentation in the central part of the cultured tissue. These changes were prominent in the vitrified group, particularly in the absence of LPA. Flow cytometry data also confirmed this observation and demonstrated the higher percent of late apoptotic and necrotic cells in the non-LPA treated group. Hence, LPA might improve the IVC condition by decreasing cell death via the MAPK/p38, PI3K/Akt, and NF-kappa B signaling pathways. Several studies reported that LPA could be a survival and anti-apoptotic factor (34). It has been suggested that in the vitrified group, due to alterations in the LPA receptor, this effect of LPA might not have been properly shown. Additional studies should prove this suggestion. 

The percentage of late apoptotic and necrotic cells significantly increased in all study groups after 7 days of *in vitro* culturing. 

This conclusion agreed with Keros et al. (40). These researchers postulated that the freezing and thawing process influenced cells and led to necrosis in stromal cells. Totally the proportions of apoptotic (early and late) cells in all study groups were significantly higher than necrotic cells. It seemed that both factors (vitrification and culture conditions) adversely affected cell survival and caused both types of cell death.

We also assessed caspase-3/7 activity. Our results confirmed that the ovarian tissue culture enhanced caspase-3/7 activity. In addition this activity was significantly higher in the vitrified group. This conclusion agreed with the findings reported by Abdi et al. (37), which indicated that the IVC of ovarian tissue and vitrification/ warming procedure enhanced the activity of caspase-3/7, which had negative effects on follicular survival and development. However, the level of caspase-3/7 activity decreased in the presence of LPA. 

## Conclusion

Both vitrification and the IVC appeared to adversely affect cell survival and resulted in cell death. We have postulated that LPA supplementation of culture medium could improve the developmental rate of follicles and act as an anti-cell death factor in non-vitrified and vitrified ovarian tissues. It could be applicable for *in vitro* maturation of ovarian tissue. 
